# Serological Evidence of Contrasted Exposure to Arboviral Infections between Islands of the Union of Comoros (Indian Ocean)

**DOI:** 10.1371/journal.pntd.0004840

**Published:** 2016-12-15

**Authors:** Koussay Dellagi, Nicolas Salez, Marianne Maquart, Sophie Larrieu, Amina Yssouf, Rahamatou Silaï, Isabelle Leparc-Goffart, Pablo Tortosa, Xavier de Lamballerie

**Affiliations:** 1 Centre de Recherche et de Veille sur les Maladies Emergentes dans l’Océan Indien, (CRVOI) Plateforme de Recherche CYROI, Sainte Clotilde, Reunion Island, France; 2 Unité Mixte de Recherche «Processus Infectieux en Milieu Insulaire Tropical» (UMR PIMIT), INSERM 1187 CNRS 9192 IRD 249 Université de La Réunion, Plateforme de Recherche CYROI, Sainte Clotilde, La Réunion, France; 3 Aix Marseille Université, IRD, EHESP French School of Public Health, EPV UMR_D 190 "Emergence des Pathologies Virales", Marseille, France; 4 French National Reference Centre for Arbovirus, IRBA, Marseille, France; 5 Cellule Interrégionale d'Épidémiologie Océan Indien (Cire OI), Institut de Veille Sanitaire, Saint Denis, La Réunion, France; 6 Programme National de Lutte contre le Paludisme, Moroni, Union of the Comoros; University of Texas Medical Branch, UNITED STATES

## Abstract

A cross sectional serological survey of arboviral infections in humans was conducted on the three islands of the Union of Comoros, Indian Ocean, in order to test a previously suggested contrasted exposure of the three neighboring islands to arthropod-borne epidemics. Four hundred human sera were collected on Ngazidja (Grande Comore), Mwali (Mohéli) and Ndzouani (Anjouan), and were tested by ELISA for IgM and/or IgG antibodies to Dengue (DENV), Chikungunya (CHIKV), Rift Valley fever (RVFV), West Nile (WNV), Tick borne encephalitis (TBEV) and Yellow fever (YFV) viruses and for neutralizing antibodies to DENV serotypes 1–4. Very few sera were positive for IgM antibodies to the tested viruses indicating that the sero-survey was performed during an inter epidemic phase for the investigated arbovirus infections, except for RVF which showed evidence of recent infections on all three islands. IgG reactivity with at least one arbovirus was observed in almost 85% of tested sera, with seropositivity rates increasing with age, indicative of an intense and long lasting exposure of the Comorian population to arboviral risk. Interestingly, the positivity rates for IgG antibodies to DENV and CHIKV were significantly higher on Ngazidja, confirming the previously suggested prominent exposure of this island to these arboviruses, while serological traces of WNV infection were detected most frequently on Mwali suggesting some transmission specificities associated with this island only. The study provides the first evidence for circulation of RVFV in human populations from the Union of Comoros and further suggests that the virus is currently circulating on the three islands in an inconspicuous manner. This study supports contrasted exposure of the islands of the Comoros archipelago to arboviral infections. The observation is discussed in terms of ecological factors that may affect the abundance and distribution of vector populations on the three islands as well as concurring anthropogenic factors that may impact arbovirus transmission in this diverse island ecosystem.

## Author Summary

Peculiarities of Island ecosystems make oceanic islands most suitable areas for analyzing how variations in local ecological conditions may impact on the intensity of arboviral transmission. The Comoros archipelago (2144 Km2) consists of four volcanic islands that are distant of 40–60 Km apart, located at the northern end of the Mozambique Channel. The three northern islands constitute the Union of Comoros that ranks amongst the least developed countries in the world. The geographic proximity to Africa exposes the Comoros archipelago to arboviral emergence as evidenced by a number of epidemics recorded in the last decades (*i*.*e*. dengue, chikungunya, and Rift Valley fever). Most interestingly, there is historical evidence that these epidemics have had contrasted courses and severity on the 3 islands of the Union of the Comoros, despite their cultural homogeneity and the frequent inter island mobility of people and livestock. Based on these observations, we conducted a serological investigation in order to delineate the risk to arboviral infection on the 3 islands of the Union of Comoros and assess whether it is relevant to all arboviral species or only to some of them. Our results confirm the differential exposure of the Comoros islands to the arboviral risk. The ecological and anthropological factors that may account for this contrasted epidemiology are discussed.

## Introduction

Vector-borne infections are mostly sensitive to environmental changes whether natural or anthropogenic. Slight variations in ecologic conditions may severely affect pathogen transmission capacity by impacting the diversity, abundance and/or behavior of vectors, their competence for transmission, together with several traits of the parasite itself [1,2]. Unraveling these factors helps understanding how vector borne diseases may express contrasted dynamics in different geographic locations and identifying drivers of emergence acting either at local, regional or large distance scales. Island ecosystems provide ideal *in natura* conditions allowing to uncouple local transmission from long distance spread [3]. The peculiarities of such ecosystems (geographic isolation, limited land surface area, low species richness and often-high levels of endemism), make oceanic islands most suitable for comprehensive entomological surveys and investigations of original host/pathogen interactions [3].

The Comoros archipelago (2144 Km2) consists of four volcanic islands that have emerged *de novo* from the South-Western Indian Ocean (SWIO) floor at the Comoros hot spot. They are distant of 40–60 Km from each other and are aligned on a Northwest to Southeast line, at the north end of the Mozambique Channel. The three northern islands constitute the Union of Comoros that ranks amongst the least developed countries in the world. The southern island of Mayotte is administered by France and exhibits significantly higher development indices.

The islands of the Union of Comoros are small and sparsely populated. The largest island (1146Km2) sheltering the capital is named Ngazidja (Grande Comore in French) and has 380 000 inhabitants. The second larger island, Ndzouani (Anjouan) (424 Km2) has 300 000 inhabitants, while Mwali (Moheli) is the smallest island (290Km2) and is home to less than 45 000 inhabitants.

The geographic proximity to Africa exposes the Comoros archipelago to arboviral emergence as evidenced by a number of epidemics recorded in the last decades. Hence, dengue, chikungunya and Rift Valley fever outbreaks flared up on the Comoros islands in first place before spreading to other islands of the SWIO. Most interestingly, there is evidence that these epidemics have had contrasted courses and severity on the 3 islands of the Union of the Comoros, despite their cultural homogeneity and the frequent inter island mobility of people and livestock.

The different exposure of the three islands to the arboviral risk is most evidenced by the last epidemics of dengue and chikungunya. In 1993, a large epidemic due to Dengue virus serotype 1 (DENV1) affected Ngazidja, with a prevalence of 26% of IgM antibodies to Dengue virus (DENV) [4]. In contrast, the outbreak was much less severe on the sister islands of Ndzouani and Mwali where a ten times lower IgM positivity rate was detected [4], and no dengue epidemic was reported at the same time on Mayotte. In March 2010, a Dengue virus serotype 3 (DENV3) epidemic was reported on Ngazidja where 1805 suspected cases were registered while the number of suspected cases was only 18 and 4 on Mwali and Ndzouani respectively, as informally reported by the Comorian Health Authorities (www.reseausega-coi.org/system/files/06-Mlindasse-DENV3_0.pdf15). Only 76 cases of dengue were confirmed on Mayotte despite an efficient surveillance system [5].

In 2004, a CHIK outbreak flared up in Lamu, Kenya [6] then spread to Ngazidja where 5202 cases were reported from January to May 2005. A sero-survey conducted on the island during the epidemic showed an attack rate of 63% with 60% IgM and 27% IgG positive samples [7] According to the Comorian Health Authorities [8], the outbreak appeared much less severe on the other Comorian islands with 207 cases reported on Ndzouani and only 1 on Mwali. On Mayotte, the epidemic curve delineated two waves [9]: the first wave (April-June 2005) was concomitant to the epidemic on Ngazidja but of minor intensity as assessed by the very low prevalence (1.6%) detected on October 2005; the second wave was however explosive: it started on early 2006 and peaked on March-April with a dramatic rise of CHIK virus (CHIKV) seroconversion rate. This second wave then expanded into a huge epidemic that involved all the SWIO islands, *i*.*e*. Madagascar, Reunion, Mauritius, and Seychelles [10]. Interestingly the second explosive outbreak completely spared the three islands of Union of Comoros where the CHIK dynamics was unimodal.

Though previous investigations suggest a differentiated impact of arboviral diseases on the three islands, the available information supporting this view are of unequal solidity with regard to the three islands. Hence, in order to get an unequivocal assessment of this point, we realized a serological investigation in the Union of Comoros in order to delineate the arboviral risk and check whether this assumption is relevant to all viral species or only to some of them.

## Materials and Methods

### Ethics statement

The present study was implemented at the initiative of Health authorities of the Union of Comoros (Ministère de la Santé, de la Solidarité et de la Promotion du Genre) in order to evaluate the impact of vector borne diseases on the general population. The National Malaria Control Program (PNLP) was identified by the Health authorities as the promoter of the study to supervise the serological investigation according to a protocol ensuring that all samples were anonymized and the study conducted ethically (#1175/MSSPG/DNS). The present study used excess sera collected from the PNLP and from private laboratories established on Ngazidja, Ndzouani and Mwali. The PNLP has approved that only an oral informed consent should be required from the participants, in agreement with the Comoros cultural norms. The consent was collected by one of the co-authors (RS), who is member of the PNLP and explained the objectives of the investigation, how it will be realized and that anonymity will be strictly respected. Anonymization was achieved before transmission to CRVOI (Reunion Island) and the only information made available to this laboratory was the age, gender and the island of origin for each sample.

### Study design and sample collection

This is a cross sectional study involving 400 individuals living on Ngazidja (n = 196), Ndzouani (88) and Mwali (116), the three main islands of the Union of Comoros. Anonymous participants were 325 individuals suffering diverse medical conditions, consulting either private laboratories of the three islands or the Surveillance Laboratory for Malaria (PNLP) located on Ngazidja. Patients consulting private laboratories had heterogeneous conditions and were mainly suffering from chronic diseases (diabetes, hypertension etc.) or occasionally fever, while PNLP patients were mostly febrile. Seventy-five healthy individuals accompanying patients also accepted to participate to the study and represented 22, 13 and 18% of all individuals enrolled on Ngazidja, Mwali and Ndzouani, respectively. The study was conducted from August 1–October 8, 2011 and was based on excess sera remaining after laboratory tests motivating the visit to the medical center were performed. The eligibility criteria were acceptance to participate to the study and age over 15 years. Sera were stored at -20°C until testing.

The number of required sera from each island was calculated taking into account expected seropositivity rates based on literature [4,7,11] in order to be able to show a difference of 20% between islands for CHIKV and DENV, and of 6% for RVFV. Hence, the minimum sample size was estimated to 100 per island for CHIKV and DENV and to 190 per island for RVFV (S1 Table). The numbers of sera actually collected and included in the present study were 196, 116 and 88 originating from Ngazidja, Mwali and Ndzouani, respectively.

### ELISA-based detection of antibodies to arboviruses

#### IgM antibodies to arboviruses

In-house immunoglobulin (Ig) M-capture enzyme immunoassays (MAC-ELISA) were used to detect serum IgM antibodies to CHIKV, DENV, WNV and RVFV. Briefly, IgM antibodies were captured with rabbit anti-human IgM antibodies (Interchim, Montluçon, France). CHIKV, DENV, WNV and RVFV antigens were prepared on Vero cells in a BSL-3 environment, precipitated and inactivated by betapropiolactone (Sigma-Aldrich, St Quentin Fallavier, France). Specific antigen binding was demonstrated by using an ascitic fluid from mouse hyperimmune to CHIKV, DENV, WNV, and RVFV, respectively and a goat anti-mouse peroxidase-labeled conjugate (Interchim). Serum samples were considered positive if the optical density at 450 nm was >3-fold the mean of negative sera with a Sunrise spectrophotometer (Tecan, Lyon, France) [12].

#### IgG antibodies to arboviruses

The screening of antibodies (IgG) against various pathogens was performed using both in house (Yellow fever virus (YFV), CHIKV and RVFV) and commercial (DENV, Tick-borne encephalitis virus (TBEV) and West Nile virus (WNV)) enzyme linked immune-absorbent assay (ELISA) protocols. Yellow fever virus, CHIKV and RVFV assays were described elsewhere [13] and relied on the use of antigen derived from whole-virion particles in non-inactivated cell culture supernatants. They were operated in a BSL-3 environment. Commercial kits were used according to the manufacturers' recommendations for detection of antibodies to DENV (PanBio^®^, Brisbane, Australia), WNV and TBEV (EuroImmun^®^, Lübeck, Germany). Positive and negative control sera were provided by the French National Reference Centre for Arboviroses or by the manufacturers. For each serological assay, a minimum of 3 positive controls was included, alongside 3 negative and 3 blank samples (normal saline) used as controls. For consistence, all samples were tested in duplicates using common serum controls (negative and positive) for all plates in a specific pathogen assay. The values of all plates for a given test were subsequently normalized according to values of negative and positive controls. In addition, results from a panel of 176 true negative samples (based on sero-neutralisation methods) were used for determining cut-off values of both in-house and commercial kits [14]. Sera with normalized absorbance values above the cut-off value (defined as [mean of normalized true negatives + two standard deviations]) were considered to be positive (Absorbance Ratio (AR)>1.1). As members of the flaviviridae (*i*.*e*. DENV, WNV, TBEV and YFV) are to a large part sero crossreactive, a more stringent criteria was applied to the data pertaining to this viral family in order to identify among the four viral species the most probable etiologic virus. The latter was defined as the virus giving the AR>1.1 plus at least 0.5 over the AR of any the 3 other flaviviruses.

### DENV sero-neutralisation tests

DENV serotyping was performed using an ELISA-format micro neutralizations test as previously described [15] In this assay, viral neutralization is not measured by the reduction of the number of lysis foci, but rather by the reduction of viral proteins produced in the plate, as detected with a spectrophotometer through an indirect immunoperoxydase antibody test. This assay has been shown to provide monotypic responses similar to the standard serotype-specific PRNT assay in serum post primary infection [16]. Briefly, viruses were grown in 96-well plates in Vero E6 monolayers in the presence of two-fold serial dilutions of serum ranging from 1/40 to 1/2560. Seven days post-infection, supernatants were removed; cells were fixed with 4% para-formaldehyde and permeabilized with 0.5% Triton X-100. Viral proteins were detected spectrophotometrically using a commercially available pan-flavivirus anti-NS5 monoclonal antibody from hybridoma H86.13 B4A supernatant, HRP-conjugated anti-mouse secondary antibodies and TMB as peroxidase substrate. For each serum and virus, the neutralization titer was the reciprocal of the highest serum dilution that inhibited virus protein production, *i*.*e*. leading to a mean OD below the positive cut-off value as determined with the corresponding control test DENV. The DENV serotype that is neutralized at the highest serum titer is referred as the *dominant serotype*. Negative controls were performed using four true negative samples (courtesy of the French National Reference Centre for Arboviruses). The four DENV serotypes used in the assay were H/IMTSSA/98/060, H/IMTSSA-MART/98-703, H87, and Dak HD 34 460, respectively [16–19] Sera with neutralizing titer ≥40 were considered positive.

### Statistical analysis

Seropositivity rates were first calculated for each arbovirus on the whole sample, then according to island, age, and clinical condition (“healthy” versus “unhealthy” individuals) and compared together using chi-square or Fisher's exact test.

## Results

From August 1^st^ to October 8^th^ of 2011, 400 excess sera were collected in private laboratories and PNLP, from 75 healthy individuals and 325 individuals suffering various clinical conditions (Sex Ratio M/F = 0.61) who sought laboratory testing for diverse medical reasons, including blood smear analysis for malaria diagnosis in febrile patients. Characteristics of the population sample according to age class, gender and living place, are provided in Table 1. A total of 196 (49.0%), 116 (29.0%), and 88 (22.0%) subjects originated from Ngazidja, Mwali and Ndzouani, respectively. Sixty seven (16.7%), 133 (33.3%), 131 (32.7%) and 69 (17.3%) sera were from children (<15years old (yo)), young adults (15–30 yo); adults (31–50 yo) and older adults (>50 yo), respectively.

**Table 1:** Distribution of the study sample according to gender, age class and island.

	<15yo	15-30yo	31-50yo	>50yo	All age classes
Ngazidja	41	52	59	44	196
Females, Males	23, 18	32, 20	36, 23	25, 19	116, 80
Mwali	4	42	51	19	116
Females, Males	3, 1	34, 8	30, 21	8, 11	75, 41
Ndzouani	22	39	21	6	88
Females, Males	9, 13	31, 8	15, 6	3, 3	58, 30
Total	62	133	131	74	400

IgG Seropositivity rates are reported on Table 2 for each arbovirus in the whole sample and according to the living Island. Data show that most individuals (75%) have been previously infected by DENV while seropositivity rates to CHIKV, RVFV and WNV were much lower (12.0%, 10.7% and 7.2%, respectively). Finally, very few individuals were seropositive for TBEV and YFV (0.7% and 0.5%, respectively). When the data generated from individuals suffering clinical conditions (n = 325) and from healthy individuals (N = 75) were analyzed separately (Table 2), the prevalence rates were not statistically different between the two subgroups for DENV, CHIKV, YFV and WNV but were found significantly higher for RVFV and TBEV in the subgroup of healthy individuals.

**Table 2:** IgG antibodies to each arbovirus according to living Island and to the status of serum donors (n = 400). The positivity criteria is a Absorbance Ratio to the cut-off value >1.1.

*Arbovirus*	Ngazidja n = 196	Mwali n = 116	Ndzouani n = 88	Total n = 400	*p*	Healthy group n = 75	Unhealthy group n = 325	*p*
	n (%)	n (%)	n (%)	n (%)		n (%)	n (%)	
CHIKV	37 (18.9)	9 (7.8)	2 (2.3)	48 (12.0)	<0.0001	12 (16.0)	36 (11.0)	0.24
RVFV	31 (15.8)	6 (5.2)	6 (6.8)	43 (10.7)	0.005	17 (22.7)	26 (8.0)	<0.001
DENV	174 (88.8)	77 (66.4)	49 (55.7)	300 (75.0)	<0.0001	54 72.0)	235 (72.3)	0.96
TBEV	3 (1.5)	0 (0.0)	0 (0.0)	3 (0.7)	0.20	3 (4.0)	0 (0.0)	<0.01
YFV	2 (1.0)	0 (0.0)	0 (0.0)	2 (0.5)	0.35	0 (0.0)	2 (0.6)	0.96
WNV	6 (3.1)	21 (18.1)	2 (2.3)	29 (7.3)	<0.0001	6 (8.0)	14 (4.3)	0.19

In addition to IgG serology that was carried out on the whole sample, there was enough residual serum to screen for the presence of IgM antibodies against DENV, CHIKV, WNV and RVFV viruses for 364 samples. Very few sera were found seropositive for IgM to DENV, WNV and CHIKV (3, 2 and 0 sera respectively) (Table 3). In contrast, higher rates of seropositivity for IgM to RVFV were recorded in the three islands with the highest rates on Mwali (10.25%), and Ndzouani (8.04%) compared to Ngazidja (2.5%) (p<0.05).

**Table 3:** IgM antibodies to DENV, WNV, CHIKV and RVFV in 364 sera collected from Grande Comore, Moheli and Anjouan.

*Arbovirus*	Ngazidja n positive /n tested (prevalence in the sample)	Mwali n positive /n tested (prevalence in the sample)	Ndzouani n positive /n tested (prevalence in the sample)	Total (prevalence in the sample)
DENV	2/ 160 (1,25%)	1/117 (0.85%)	0/87 (0%)	3/364 (0.82%)
WNV	0/160 (0%)	2/117 (1.70%)	0/87 (0%)	2/ 364 (0.55%)
CHIKV	0/160 (0%)	0/117 (0%)	0/87 (0%)	0/ 364 (0%)
RVFV	4/160 (2.5%)	12/117 (10.25%)	7/87 (8.04%)	23/ 364 (6.31%)

### IgG seropositivity rates to DENV

A total of 300 sera tested positive through ELISA for IgG antibodies to DENV. This consisted of 174, 77 and 49 sera from Ngazidja, Mwali and Ndzouani, corresponding to 88.8%, 66.4% and 55.7% seropositivity rates, respectively. The difference in seropositivity according to island was highly significant (p<0.0001).

As several dengue epidemics hit the Comoros archipelago during the last 70 years, we typed sera against each of the 4 DENV serotypes using the ELISA-format micro neutralization test derived from the plaque reduction neutralization test (PRNT). A subgroup of 90 sera testing ELISA positive for DENV was randomly selected from persons aged less than 50. Sera from individuals over age 50 were not considered for this test since interpretation of the neutralization profiles is more complicated in older age classes which are more likely to have experienced sequential infections by different DENV serotypes, thus inducing stronger heterotypic DENV neutralization responses. Four samples testing ELISA negative were used as controls. Twelve sera tested negative by the ELISA-format micro neutralization test despite being positive through ELISA to DENV antigen, and were not further considered. Seventy-eight sera (54, 18 and 6 originating from Ngazidja, Ndzouani and Mwali, respectively) had individual titers over 40 to one or several DENV serotype(s) and were considered positive to the reactive serotype (Table 4). The mean titer of reactivity was 1093, 835, 566 and 718, for serotypes 1 to 4, respectively. Noteworthy, only few sera (14 out of 78), reacted with one single serotype (Table 5) If one considers only the dominant serotypes (*i*.*e*. the serotype identified at the highest titer by each serum sample), the most frequently recognized serotypes were DENV serotypes 1 and 3, particularly on Ngazidja (Table 4). Noteworthy, serotype 3 was mostly recognized in sera from young persons, and serotype 1 from older individuals.

**Table 4:** Number of sera with neutralizing antibodies to DENV serotypes 1–4 according to (i) the neutralized serotype(s), (ii) the dominant serotype, (iii) the Island and (iv) the age class. Presented data concern a subset of 90 randomly selected DENV ELISA positive samples. (* Numbers may not add up as one individual serum may react with more than one serotype).

DENV Serotype	Serotype	Serotype	Serotype	Serotype
	1	2	3	4
i) Number* of sera with neutralizing antibodies to the indicated DENV serotype:	57 (63.3%)	47 (52.2%)	68 (75.6%)	44 (48.9%)
ii) Number* of sera with neutralizing antibodies to the indicated serotype considered as dominant serotype:	35 (38.9%)	21 (23.3%)	31 (34.4%)	14 (15.6%)
iii) Island:				
Grande Comore (Ngazidja)	25	15	25	7
Moheli (Mwali)	8	4	4	6
Anjouan (Ndzouani)	2	2	2	1
iv) Age class:				
<15yo	8	6	16	1
15-30yo	12	4	8	4
30-50yo	15	11	7	9

**Table 5:** Neutralizing antibodies for DENV serotypes 1–4. Reactivity profile according to the number of seroneutralized serotypes detected in 78 out of 90 randomly selected DENV ELISA positive samples.

Island of origin and age class	Grande Comore (Ngazidja)	Moheli (Mwali)	Anjouan (Ndzouani)	Total tested	<15yo	15-30yo	30-50yo
Number of sera with neutralizing antibodies to DENV serotypes 1–4	54	18	6	78	23	22	33
Number of sera with neutralizing antibodies to DENV according to the number of reactive serotypes							
1 serotype	5 (9.2%)	6 (33.3%)	3 (50.0%)	14	4 (17.4%)	6 (27.3%)	4 (12.1%)
2 serotypes	17 (31.5%)	5 (27.8%)	0 (0.0%)	22	10 (43.5%)	7 (31.8%)	5 (15.1%)
3 serotypes	12 (22.2%)	3 (16.7%)	1 (16.6%)	16	6 (26.1%)	2 (9.1%)	8 (22.2%)
4 serotypes	20 (37.0%)	4 (22.2%)	2 (33.2%)	26	3 (13.0%)	7 (31.8%)	16 (48.5%)

### IgG seropositivity rates to CHIKV, RVFV and WNV

A total of 48 sera were positive for IgG antibodies to CHIKV. A significant difference was observed according to the sampled island (p = 0,0001), with a seropositivity rate higher on Ngazidja (18.9%) than on Mwali (7.8%) and Ndzouani (2.3%). No difference in seroprevalence was observed according to age.

Forty-three sera (10.7%) were positive for IgG antibodies to RVFV with seropositivity rates being again statistically different according to the sampled island (p<0.001). Sera from Ngazidja, Mwali and Ndzouani displayed IgG seropositivity rates to RVFV of 15.8%, 5.2% and 6.8% (see Table 2), respectively. No significant difference in seroprevalence was observed according to age, although it tended to be higher in adults >50 yo (18.9%) than in the three other age groups (10.4%, 9.8% and 7.6%, respectively). Finally, 29 sera were positive for IgG antibodies to WNV by ELISA, with most of them (21/29) originating from Mwali (Table 2). Using the most stringent criteria to discriminate between infections by members of flaviviridae family (See Material and Methods section), 21 among these 29 sera were unambiguously positive for WNV (S1 Table), 7 sera yielded ambiguous results between WNV and DENV and one serum between WNV and TBEV. Interestingly, most (15/21) sera unambiguously testing positive for WNV still originated from Mwali. This was also the case for 5 out of the seven sera with ambiguous positivity to both DENV and WNV (S1 Table). These results support the selectivity of WNV infection on Mwali.

### IgG antibodies to tick-borne encephalitis and yellow fever virus

Three sera were positive for TBEV and two for YFV and they were all originated from Ngazidja. Altogether, among the 400 sera composing the serobank, 338 (84.5%) reacted with at least one of the investigated arboviruses. The seropositivity rate to at least one arbovirus showed a strong association with age (p<0.0001) since it was 68.7%, 81.2%, 90.1%% and 95.7% in the four successive age groups (<15yo; 15-30yo; 31-50yo; >50yo), respectively.

## Discussion

IgG antibodies to arboviruses are long lasting signatures of the arboviral outbreaks that have been hitting inhabitants of the Comoros Islands for decades. Almost 85% of sera investigated in the present study reacted with at least one arbovirus and the seropositivity rate increased with age, stressing the intense and long lasting exposure of the Comorian population to arboviral risk. As demonstrated by the almost negative results for IgM antibodies, this study was conducted during an inter-epidemic phase for DENV, CHIKV and WNV infections. However for RVFV, we had evidence of recent or current circulation of RVFV on the whole country though mostly on the two southern islands of the Union of Comoros. The two subgroups composing the whole sample used in the study (*i*.*e*. “not healthy” individuals consulting private or public laboratories for various medical reasons n = 325; and “healthy” individuals (n = 75) had prevalence rates not statistically different for DENV, CHIKV, YFV and WNV but significantly higher for RVFV and TBEV in the control group. At the very least, this result argues that our data were not biased towards more detection of arboviral infections in the “not healthy” individuals (especially the febrile ones) compared to “healthy” individuals. Our data also indicate that the most significant risks pertain to DENV, CHIKV, RVFV and WNV infections, with DENV exposure clearly appearing as the most prominent. A strict criterion was used to define positive sera based on a panel of true negative samples that were confirmed by sero neutralization. Noteworthy, the use of the more stringent criteria to identify the likely infecting virus among the flaviviruses (*i*.*e*. DENV, WNV, TBEV and YFV), yielded essentially similar results (see S1 Table). Titration of neutralizing antibodies to the four DENV serotypes indicates that most sera react with more than one serotype, an additional confirmation of the iterative epidemics bouts hitting these islands. Sera were most frequently reactive with DENV1 and DENV3 and less frequently with DENV2 and DENV4. These results are in keeping with the history of dengue epidemics on the archipelago during the last seventy years: DENV1 epidemics was recorded in 1948 and 1993, DENV2 in 1984 [20] and DENV3 in 2010 [21] To our knowledge, serotype 4 has never been reported as causing an epidemic in the Indian Ocean region though some uncertainty remains with regard to serotypes that have been circulating during the 1948 and 1984 epidemics in Comoros [22]. Yet, neutralizing antibodies to DENV4 were detected in the present series in over half of titrated sera (44/78). As for sera neutralizing multiple serotypes, this DENV4 positivity may reflect a mere cross reactivity. However, 14 sera expressed the highest neutralizing titer to this serotype 4 and these sera were mostly (9 out of 14) from adults aged over 30. It is therefore probable that DENV4 has been circulating at low rate, presumably several decades ago.

Considering all DENV serotypes, our data bring in further evidence that human populations living on Ngazidja have been more exposed to dengue epidemics than those living on Mwali or Ndzouani. In the present study, IgG antibodies to DENV were detected in 89%, 66% and 56% of sera from individuals living in Ngazidja, Mwali and Ndzouani, respectively, the difference in seropositivity rates being highly significant. In addition, most sera reacting with only one DENV serotype were from Mwali and Ndzouani (9/14) whereas most sera from Ngazidja reacted with multiple serotypes and over one third of them with all four DENV serotypes. These data are in keeping with previous reports showing that during the epidemics of 1993, rates of IgG positivity were 83% on Ngazidja, 40% on Mwali and 22.5% on Ndzouani [4].

It is likely that compared to Ngazidja, the relative preservation from arboviral risk of the southern islands of the archipelago also holds true for Mayotte. In that island, a cross-sectional serologic survey conducted in 2006 during an inter-epidemic period for dengue, showed a rate of DENV-specific IgG antibodies of 22.7% with an age-specific prevalence peaking at 38.8% in the 25–54 years age group while the 2–14 age group was almost completely preserved [20]. Interestingly, Comorian native inhabitants aged 15 years or over, who had immigrated to Mayotte, were around ten-times more affected than individuals of the same age born in Mayotte [20].

The seroneutralisation analysis carried out in the present study on a subset of dengue ELISA positive samples indicates that almost all of these sera actually contained antibodies neutralizing DENV, with two thirds neutralizing 3 or 4 serotypes. Hence, a large fraction of the population (>40%) could be considered as currently protected against dengue. This could explain the long interval (several decades) between two epidemic bouts, allowing immunologically naive individuals (mainly young persons) to accumulate into the pool of susceptible until the next dengue outbreak flares up. These periodic bursts do not preclude a low level of DENV transmission in the Union of Comoros during inter-epidemic periods; however proof of such low-rate transmission is missing as most dengue like syndromes are not investigated in this resource limited country.

With regard to Chikungunya virus, our results indicate a similar trend toward a more severe exposure of Ngazidja to this arbovirus compared to the two other islands. Our study has detected IgG seropositivity rates to CHIKV of 18.9% on sera from Ngazidja and 5.8% and 2.3% on sera from Mwali and Ndzouani, respectively. Surprisingly, the seropositivity rate on Ngazidja appeared rather low compared to the attack rate of 63% (60% IgM antibodies, 27% IgG) reported on this island during the large epidemic of Chikungunya in 2005 [7] In fact, the IgG seropositivity rates reported by Sergon and co-workers [7] and the one detected in the present study are relatively close and the contrast is mainly created by the very high level of IgM reported during this epidemic. This apparent discrepancy suggests either that IgM positivity to CHIKV was overestimated by Sargon *et al*. or that a large fraction of IgM positive individuals actually failed to efficiently switch into high titres and long lasting IgG antibodies to CHIKV. Few data have been reported so far in the literature on the long-term persistence of antibodies to CHIKV following infection. However, almost 10% of seropositive sera from individuals infected on Reunion Island during the 2006 epidemics reverted to ELISA negative within 3 years after the epidemics (A. Michault, personal communication). In Thailand, nineteen years after a CHIKV outbreak occurred, over one third of 111 former patients had neutralizing antibodies reacting against CHIKV antigen [23]

In addition to showing maximal exposure of Ngazidja inhabitants to DENV and CHIKV, our study brings in the first serological evidence for RVFV circulation amongst humans on the islands of the Union of Comoros. This was demonstrated not only by high rates of IgG seropositivity to the virus but also by the detection of IgM seropositivity indicating recent or current circulation of the virus mainly through pauci- or a-symptomatic infection. Rift Valley fever is a vector borne zoonotic disease present in Africa, the Middle East, and Madagascar [24–28]. Our data reveal higher IgG seropositivity rates in Ngazidja but higher IgM seropositivty rates in Mwali and Ndzouani. These results confirm the only suspicion going back to 2007 when an autochthonous clinical case of RVFV was detected in a child from Ngazidja admitted at Mayotte’s hospital with a 2-month history of severe encephalitis [28]. They are also coherent with the demonstration of an active circulation of the virus in Comorian livestock [29,30]. With regard to Mayotte, an investigation carried out on 2007–2008 on dengue-like syndromes, testing negative for *Plasmodium* spp., CHIKV, and DENV, revealed 4.5% positive cases, suggestive of recent RVFV infection, *i*.*e*. presence of viral RNA or IgM [31]. A sero-survey study (2011) conducted also on Mayotte on 1420 individuals showed an overall seroprevalence of 3.5% for RVFV antibodies in the general population aged over 5 years. Altogether these results indicate active circulation, though mostly clinically insidious, of RVFV in humans in the four islands of the Comoros archipelago.

Finally, our study is the first reporting on WNV infection in the Comoros archipelago. Several studies have shown active circulation of WNV on Madagascar [32–34] and Seychelles [35], and the virus has been isolated at several occasions from birds in Madagascar [33]. Interestingly the island of Mwali clustered 75% of sera testing positive to this flavivirus in our study, indicating that among the Comorian population, inhabitants of Mwali bear the maximal risk of WNV infection. The use of stringent criteria to discriminate true WNV infection, as well as the observation that most seropositive sera clustered in the smallest island of the Union of Comoros are strong arguments against a mere cross reactivity with other flaviviruses. However, the formal proof would require testing for neutralizing antibodies.

Thus, our study highlights the distinct exposure of the different Comorian islands to arboviral risk and one may address the question on which factors could account for such peculiarity. Geographic, ecologic, entomologic and anthropogenic factors are most likely the determinants of such heterogeneity for which we propose several non-exclusive hypotheses.

### Geographical and anthropogenic factors

Ngazidja, the closest to the African continent among the archipelago islands, is directly exposed to the East African coast, a hot spot for the emergence of zoonotic and vector borne diseases [36] Moreover, Ngazidja is the entry point for active trade with continental African countries conveying pathogens and vectors. For example, the bilateral trade bill established in 2000 with Tanzania had allowed importation of cattle to Ngazidja and was soon followed by the emergence of an epidemic of theileriosis on this island likely due to the introduction of the infected tick vector [37,38]. Similarly, the higher IgG seropositivity rates to CHIKV, DENV and RVFV measured on Ngazidja in the present study may simply reflect this geographical proximity. However, the higher IgM positivity rates to RVFV in sera from Mwali is in keeping with the fact that livestock was mostly infected on Mwali [29,30].

### Geological and entomological factors

Comorian islands are volcanic islands spanning along a South/North line which ages range from 7.7–15 yo in the eldest Mayotte to 0.1–0.5 yo in the youngest Ngazidja [3]. These stretched ages impact their geologic structure and shape the ecologic conditions that prevail on these islands. For instance, Ngazidja has a very shallow soil layer, which cannot hold water, while the other islands, geologically older, support more advanced soil lateritisation that allows better persistence of surface water. The absence of perennial river on Ngazidja due to the highly porous soil imposes rainfall water to be stored in catchment tanks, creating anthropogenic conditions that are conducive to the proliferation of mosquito larvae. Subtle differences may exist between mosquito populations in the different islands. For instance, several mitochondrial haplotypes displaying some level of geographical structuration have been characterized among natural populations of *Ae*. *aegypti* sampled throughout other islands of the South West Indian Ocean and these haplotypes may be associated with distinct morphological and ecological traits [39]. Such information, as well as studies on the vector competence of these distinct lineages towards CHIKV and DENV are not available on the Union of the Comoros and should help understanding the human serological figures highlighted here.

The present study shows that Mwali is clearly more exposed to WNV infection than the two sister islands. It is unlikely that this peculiarity relies on significantly different exposure to the bites of *Culex* spp., the usual vectors of WNV. It rather suggests, if one considers the major role played by birds in the transmission of this virus, some specificities of Mwali with regard to local birds populations. In fact, Mwali is known to host the major seabird reproduction colonies of the whole Comorian islands [40]. Further investigation of migratory routes together with an analysis of the serological status of both migratory and local bird populations will help clarifying this issue.

The main limitation of our investigation is in the study design, which for feasibility reasons was based on the use of excess sera collected from laboratory consultants. Although enrolled participants reside in either of the three main islands, they were not representative of the whole Comorian population. However our results are in keeping with the scarce studies conducted so far in this country indicating an intense exposure of all islands of the Union of Comoros to arboviral infections with salient disparities among islands with regard to some arboviruses. They stress the importance of Ngazidja as the potential entry point for arboviral infections to the whole south Western Indian Ocean region and raise interesting working hypothesis to account for the local specificities of arboviral epidemiology in this tropical island ecosystem. Controlled studies are needed to identify risk factors to various arbovirus infections to which the population of the different islands are exposed.

## Supporting Information

S1 Table Identification of the dominant seroreactive flavivirus in the whole sample according to the living island, Union of Comoros (n = 400). The dominant seroreactive flavivirus is the one with an AR>1.1 plus 0.5 higher than the data of other flaviviruses.
(DOC)

S1 Checklist STROBE Statement—Checklist of items that should be included in reports of *cross-sectional studies*. (DOC)
