# Supplementary material for: Serological Evidence of Contrasted Exposure to Arboviral Infections between Islands of the Union of Comoros (Indian Ocean)
Source: PLoS Negl Trop Dis. 2016 Dec 15;10(12):e0004840. doi: 10.1371/journal.pntd.0004840 (PMC5157944; doi:10.1371/journal.pntd.0004840)
Supplement: S1 Table — The dominant seroreactive flavivirus is the one with an AR>1.1 plus 0.5 higher than the data of other flaviviruses. (DOC) [file pntd.0004840.s001.doc]

|  | Ngazidja  (n=196) | | Mweli (n=116) | | Nzwani (n=88) | | Total  (n=400) | | p |
| --- | --- | --- | --- | --- | --- | --- | --- | --- | --- |
|  | n | % | n | % | n | % | n | % |  |
| DEN | 172 | 87.8 | 72 | 62.1 | 45 | 51.1 | 289 | 72.25 | <0.0001 |
| TBE | 3 | 1.5 | 0 | 0.0 | 0 | 0.0 | 3 | 0.7 | 0.20 |
| YF | 2 | 1.0 | 0 | 0.0 | 0 | 0.0 | 2 | 0.5 | 0.35 |
| WNV | 4 | 1.5 | 15 | 12.9 | 2 | 2.3 | 21 | 5.0 | <0.0001 |
